# Supplementary material for: Identifying Potentially Climatic Suitability Areas for Arma custos (Hemiptera: Pentatomidae) in China under Climate Change
Source: Insects. 2020 Oct 4;11(10):674. doi: 10.3390/insects11100674 (PMC7600814; doi:10.3390/insects11100674)
Supplement: Supplementary file 1 [file insects-11-00674-s001.pdf]

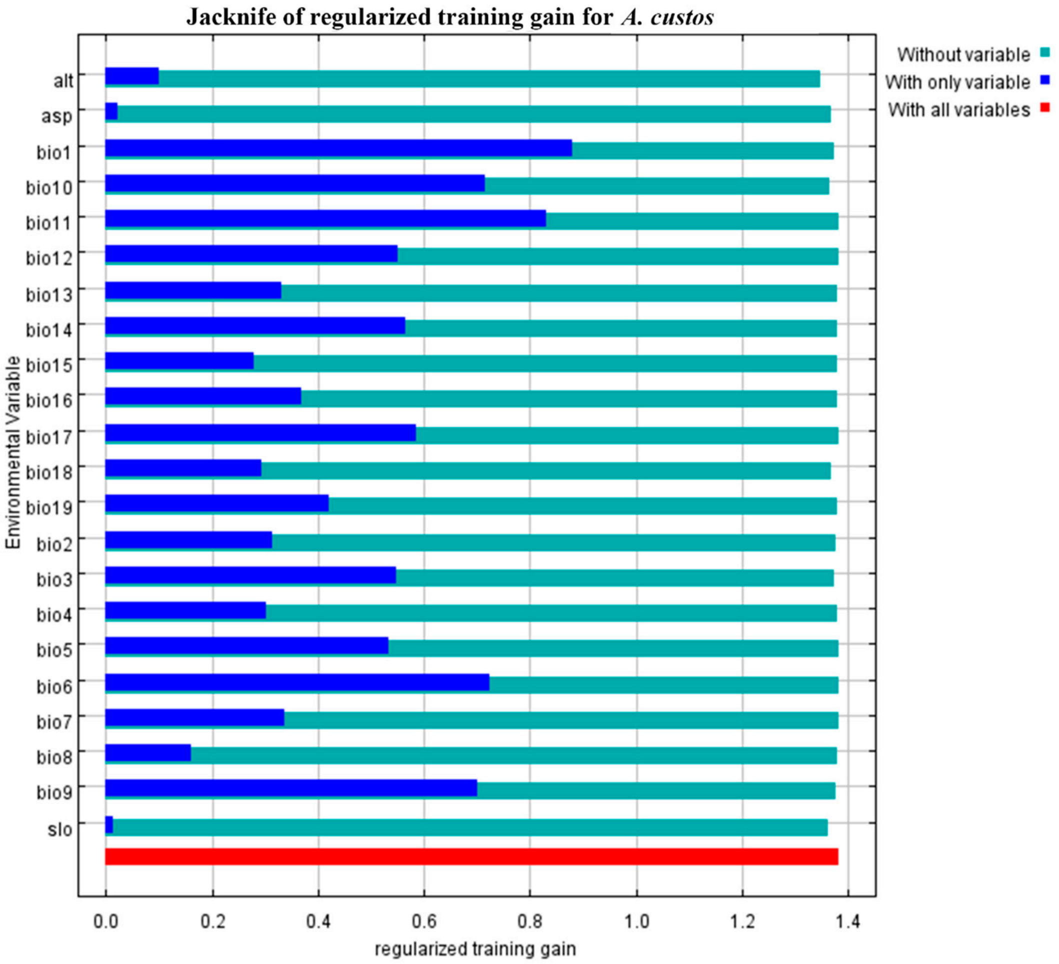

Figure S1. Jacknife for A. custos.

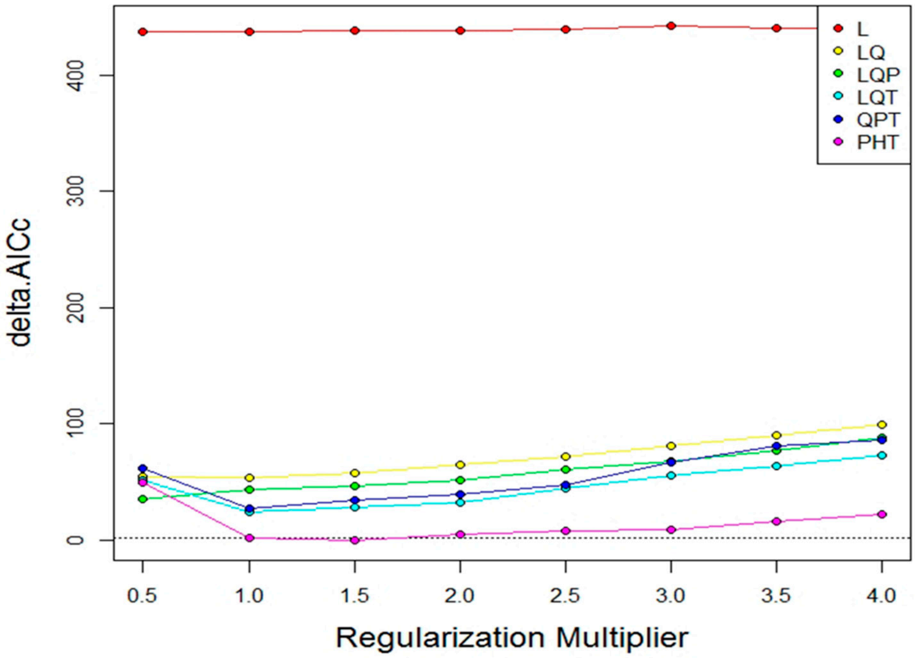

Figure S2. Delta AICc.

Table S1-1. Occurrence Data from field collection

longitude	latitude	sampling sites
126.661665	45.742367	Heilongjiang
126.549572	43.837883	Jilin
123.431383	41.836175	Liaoning
111.76629	40.81739	Inner Mongolia
116.407395	39.904211	Beijing
114.468665	38.037057	Hebei
112.711059	38.731402	Shanxi
113.41233	38.08562	Shanxi
113.657841	37.786653	Shanxi
112.548879	37.87059	Shanxi
112.734174	38.416663	Shanxi
112.151212	35.268314	Henan
112.414738	35.486029	Henan
111.669928	35.297386	Shanxi
111.007529	35.026412	Shanxi
117.020359	36.66853	Shandong
102.485646	35.848586	Qinghai
104.926337	33.392211	Gansu
105.742203	33.750477	Gansu
108.222154	34.163621	Shaanxi
113.625328	34.746611	Henan
113.927016	35.303136	Henan
114.20509	35.14189	Henan
114.392393	36.097577	Henan
114.821348	34.822211	Henan
112.789534	34.72722	Henan
112.844517	34.16703	Henan
113.609286	33.437877	Henan
112.360026	32.520805	Henan
114.091023	32.146984	Henan
117.284903	31.861121	Anhui

118.763232	32.061707	Jiangsu
104.1160498	30.68489071	Sichuan
110.800841	31.034808	Hubei
110.490346	31.319325	Hubei
106.630154	26.647661	Guizhou
105.697472	28.590337	Sichuan
107.545049	25.822132	Guizhou
107.465407	27.749055	Guizhou
104.895467	25.09204	Guizhou
107.323077	25.831955	Guizhou
106.592108	26.838926	Guizhou
106.927389	27.725654	Guizhou
108.91468	27.243012	Guizhou
108.839557	27.69965	Guizhou
106.470715	26.556079	Guizhou
105.770402	26.057362	Guizhou
104.727418	27.123079	Guizhou
105.291644	27.283955	Guizhou
106.825644	28.133583	Guizhou
106.400342	27.79165	Guizhou
105.382715	26.777645	Guizhou
108.81606	27.173887	Guizhou
106.099617	25.178422	Guizhou
106.979524	26.453154	Guizhou
104.957831	26.547904	Guizhou
107.234703	26.584666	Guizhou
112.98381	28.112449	Hunan
117.813807	28.000012	Jiangxi
119.481504	28.448772	Zhejiang
120.299402	30.419046	Zhejiang
119.281164	29.474871	Zhejiang
118.154533	29.255214	Zhejiang
102.832892	24.880095	Yunnan

102.4976	25.221935	Yunnan
104.394158	23.012915	Yunnan
100.79715	22.008811	Yunnan
118.785468	27.526232	Fujian
118.177708	26.641769	Fujian
87.616848	43.825592	Xinjiang
94.697074	43.254978	Xinjiang
93.016625	43.598763	Xinjiang
81.527454	43.977138	Xinjiang
82.51181	43.800247	Xinjiang
82.980317	46.745364	Xinjiang
88.141253	47.844924	Xinjiang
86.174633	41.725891	Xinjiang
114.109497	22.396428	Hong Kong
120.960515	23.69781	Taiwan
118.8940104	47.697848	Shaanxi
133.6375314	33.7432238	Gansu
116.407395	39.904211	Beijing
115.46459	38.874434	Hebei
118.180194	39.630867	Hebei
117.96275	40.952942	Hebei
116.838835	38.304477	Hebei
115.670177	37.73892	Hebei
114.886335	40.767545	Hunan
112.92392	35.797997	Shanxi
112.414738	35.486029	Shanxi
110.473726	36.76414	Shanxi
110.9033092	37.4340621	Shanxi

Table S1-2 Occurrence Data from GBIF Database

longitude	latitude
127.6084018	37.7829087
125.7516069	38.5023428
13.4666667	55.7
13.5957692	55.9902572
4.9514271	53.2501844
6.1560311	53.4785962
4.469936	50.503887
6.129583	49.815273
8.9425	55.0183333
15.0777111	54.9957299
10.451526	51.165691
5.928	43.124228
-9.25	38.9166667
-8.9409302	39.6182144
-3.7037902	40.4167754
2.1734035	41.3850639
2.5126761	41.7758538
2.2665964	41.7391536
12.56738	41.87194
8.4185735	45.3202272
8.9370629	44.5322241
8.946256	44.4056499
9.1582069	45.1847248
9.8787674	46.1698583
11.2621219	45.9914453
11.1217486	46.0747793
11.690976	45.7623333
13.6201754	45.9401812
11.2186396	44.5967607
11.2486208	43.7710513

13.3666667	41.8833333
13.7289167	42.1920119
14.4936511	41.6308147
14.2681244	40.8517746
16.3324483	38.5783005
17.315819	49.66843
13.9929823	50.0561694
16.7559549	49.3445311
16.6068371	49.1950602
16.6446893	48.8446246
16.8825168	48.75314
17.4027197	49.2750985
14.5824274	49.9015169
28.468217	49.233083
14.4344127	50.0707054
20.4973517	48.6302631
18.1571098	47.9881643
18.7408001	49.2194498
9.555373	47.166
15.8726231	48.8490309
16.52796	47.84637
19.503304	47.162494
14.5057515	46.0569465
16.3936719	46.68518
28.468217	49.233083
15.3002237	45.4611763
16.3429113	46.6516758
14.3836145	46.019507
15.981919	45.8150108
16.4267558	43.5470909
15.8724103	46.160492
20.5252702	42.0512389

17.9070412	44.9764185
18.4130763	43.8562586
21.1115228	45.0209346
20.4683341	44.7621061
20.6527992	42.3998287
24.96676	45.943161
26.1025384	44.4267674
28.2743836	45.2852164
28.369885	47.411631
25.5507147	41.8504028
21.4279956	41.9973462
17.0621077	49.7036967
13.910154	46.2679088
35.243322	38.963745
26.734625	41.839142
26.643187	41.964762
27.365646	41.402742
29.0609636	40.1885281
31.608209	40.732541
16.4872918	48.2194843
27.4295624	38.6140337
29.9408089	40.7654408
32.8597419	39.9333635
37.2886359	41.12724
19.145136	51.919438
24.603189	56.879635
23.881275	55.169438
27.953389	53.709807
31.16558	48.379433
22.287883	48.6208
24.029717	49.839683
24.711117	48.922633

13.2309287	50.2203622
25.594767	49.553517
28.468217	49.233083
30.5234	50.4501
34.7981	50.9077
31.28935	51.4982
32.059767	49.444433
34.551417	49.588267
30.7233095	46.482526
31.994583	46.975033
35.139567	47.8388
32.616867	46.635417
34.102417	44.952117
36.230383	49.9935
39.307815	48.574041
37.80285	48.015883
105.318756	61.52401
31.2741928	58.5255698
27.772222	57.749722
20.4522144	54.7104264
61.9316226	59.007735
49.0660806	55.8304307
35.8384768	34.4381179
48.0599345	46.3588045
44.5133034	48.708048
48.2890907	42.0674251
38.1644618	44.8715341
38.140311	45.2455578
38.987221	45.0392674
20.4130631	44.7615346
82.9357327	55.0083526
84.9924506	56.5010397

83.8482546	55.545305
57.4155949	57.0119143
83.7697832	53.3547792
82.7806142	52.4934588
86.920381	51.657116
68.2635228	58.200024
73.3242362	54.9884804
104.3050183	52.2869741
95.6260172	51.8872669
92.8932476	56.0152834
106.1051454	50.3901526
113.4711906	52.0515032
115.5499151	51.7561174
140.7314709	53.1423901
135.0662599	48.5027313
132.950623	43.7623963
136.9002967	50.1998375
141.8559579	46.678613
142.7347556	46.9641127
145.8752778	44.1491667
158.269907	57.722425
43.356892	42.315407
47.576927	40.143105
49.5891233	37.2682177
49.8624059	37.3596062
82.6017244	49.9749295
74.5697617	42.8746212
104.4141114	43.5685206
127.9784585	37.6639976
138.1942432	36.6485496
135.881652	34.273105
138.938598	34.856636

139.0400499	35.4134107
143.691	42.915
141.496356	43.0564977
130.8191722	32.4827531
13.5	55.7
10.3	52.5
13.4	52.4
10.7	53.9
7.8	49.5
11.7	48.2
10	51.6
10.8	54
12.8	56.2
10.3	52.8
10.4	45
12.7	52.4
16.3	58.1
13.5	55.7
10.5	52.4
12.9	55.8
13.4	52.5
12.1	53.9
16.3	59
26.9	58.3
13.6	55.7
13	55.4
12.9	55.8
9.8	52.7
16.8	48
14.6	51.3
8.2	50.4
7.8	51.4

7.2	51.7
13.5	55.7
10.3	50
13.4	55.9
7	51.7
13.7	51.2
10.8	50.1
10.3	52.5
7	51.7
12.6	51.5
12.2	51.4
13.5	51.4
128.1	37.5
12.6	48.2
12.5	51.2
10.1	52.7
6.7	53.6
12.3	51.4
7	51.7
12.2	50.4
9.5	48.9
13.5	51
13.9	51.2
9.8	52.7
13.9	51.2
13	50.8
14	51
11.3	47.4
10.8	51.6
9.9	52.7
9.6	48.9
7.1	50.9

6.2	50.9
14.3	56
12.5	50.6
13.5	55.7
13.6	55.7
16.4	56.7
10	54.3
13	55.8
12.8	50.9
13.6	55.7
14.7	51.3
13	51.4
13.6	55.7
13.2	55.7
14.6	51.3
13.6	55.6
13.6	55.7
13.4	55.7
14.7	51.3
11.6	49.8
13.1	51.5
13	51.4
13.5	55.7
13	50.9
51.1	51.1
55.8	55.8
48.3	48.3
55.7	55.7
51.2	51.2
50.9	50.9
51.4	51.4
50.9	50.9

50.7	50.7
51.4	51.4
51.2	51.2
51.1	51.1
51.3	51.3
50.9	50.9
51.3	51.3
50.9	50.9
50.9	50.9
51.4	51.4
39.8	39.8
51.3	51.3
49.6	49.6
141.5	39.6
141.7	39.3
141.7	40
5.4	52.1
6.3	49.8
140.1	36.3
141.5	39.3
141.5	40.3
140.9	39.6
8.9	55
140.3	36.2
141.6	39.3
5.5	52.2
141	40
4.8	51.6
15.1	55
6.1	52.7
140.8	39.5
6.3	49.5

14.2	51.4
14.2	51.1
6.7	50.6
139.6	35.9
11.5	47.3
11.4	47.3
11.7	47.3
11.6	47.3
13.9	48.5
14.1	47.9
12.6	51.4
9.8	53.3
8.7	49.4
9	47.7
12.7	51.3
14.8	51.4
14.3	48.2
14.4	48.2
14.5	51.2
14.6	51.2
12.4	51.3
16.3	48.1
12.3	51.3
14.2	48.3
14.5	51.3
14.5	51.2
14.4	51.1
14.4	51.3
14.4	48.3
14.3	48.3
12.2	51.4
14.1	50.9

12.6	51.4
12.8	51.2
16.6	52.9
14.4	48.1
12.6	51.3
12.2	51.4
12.8	51.5
12.4	51.3
14.3	48.4
15.7	48.6
7.1	50.7
132	43.2
13.7	48
14.1	48.2
16.2	48
12.8	46.8
14.4	48
14.3	48.3
15.4	47.1
16	47.1
14.1	48.1
0.9	49.2
16.2	47.7
15.9	47.9
14.3	48.3
16.4	48.3
16.3	48.3
7.4	50.2
16.5	48.2
16.1	47.9
7.1	50.7
7	47.5

16.6	47.7

Table S2. The list of original environmental variables from WorldClim

Code	Environmental variables
bio1	Annual Mean Temperature
bio2	Mean Diurnal Range (Mean of monthly (max temp - min temp))
bio3	Isothermality (bio2/bio7) (×100)
bio4	Temperature Seasonality (standard deviation ×100)
bio5	Maximum Temperature of Warmest Month
bio6	Minimum Temperature of Coldest Month
bio7	Temperature Annual Range (bio5-bio6)
bio8	Mean Temperature of Wettest Quarter
bio9	Mean Temperature of Driest Quarter
bio10	Mean Temperature of Warmest Quarter
bio11	Mean Temperature of Coldest Quarter
bio12	Annual Precipitation
bio13	Precipitation of Wettest Month
bio14	Precipitation of Driest Month
bio15	Precipitation Seasonality (Coefficient of Variation)
bio16	Precipitation of Wettest Quarter
bio17	Precipitation of Driest Quarter
bio18	Precipitation of Warmest Quarter
bio19	Precipitation of Coldest Quarter
alt	elevation
slo	slope
asp	aspect

Table S3-1 PCA Results Summary. The bold number represents all contributions of the first five principal components, accounting about 86% of total contributions.

	PC1	PC2	PC3	PC4	PC5	PC6	PC7	PC8	PC9	PC10
Standard deviation	2.8852026	2.3059705	1.6308011	1.23923985	1.08459688	0.93718842	0.79664022	0.73860777	0.60869318	0.416685776
Proportion of Variance	0.3783815	0.2417045	0.1208869	0.06980525	0.05347047	0.03992373	0.02884707	0.02479734	0.01684125	0.007892138
Cumulative Proportion	0.3783815	0.6200861	0.7409730	0.81077824	**0.86424872**	0.90417245	0.93301952	0.95781686	0.97465811	0.982550244

PC11	PC12	PC13	PC14	PC15	PC16	PC17	PC18	PC19	PC20	PC21	PC22
0.4007369	0.3474903	0.1888573	0.1828489	0.1234001	0.0784456	0.0749777	0.0556967	0.0522371	0.0217000	1.222336e-02	6.784287e-08
0.0072995	0.0054886	0.0016212	0.0015197	0.0006921	0.0002797	0.0002555	0.0001410	0.0001240	0.0000214	6.791387 e^{-06}	2.092116 e^{-16}
0.9898497	0.9953384	0.9969596	0.9984793	0.9991715	0.9994512	0.9997067	0.9998477	0.9999718	0.9999932	1.000e^{+00}	1.000e^{+00}

Table S3-2 Component Matrix of PCA. The following shows variables with highest correlation coefficients selected from the five principal components.

	PC1	PC2	PC3	PC4	PC5
Environmental variables	bio1	bio12	bio13	alt	slo
	bio11	Bio17	bio15	bio8	asp
	bio6	Bio14	bio16	bio5	bio19

Table S4. Correlation analysis of environmental variables

	alt	asp	1	10	11	12	13	14	15	16	17	18	19	2	3	4	5	6	7	8	9	slo
alt	1																					
asp	-.007	1																				
1	-.108	-.014	1																			
10	-.242	-.015	**. 936**	1																		
11	-.048	-.014	**. 979**	**. 849**	1																	
12	-.060	. 026	. 085	-.056	. 154	1																
13	. 001	. 027	. 182	. 079	. 220	**. 905**	1															
14	-.206	. 014	-.189	-.322	-.098	. 534	. 200	1														
15	. 293	. 003	. 355	. 382	. 303	-.056	. 257	-.601	1													
16	-.000	. 027	. 165	. 057	. 208	**. 931**	**. 994**	. 237	. 213	1												
17	-.198	. 015	-.164	-.300	-.072	. 589	. 254	**. 991**	-.587	. 291	1											
18	. 028	. 020	-.061	-.153	-.024	. 847	**. 802**	. 352	. 048	**. 822**	. 392	1										
19	-.129	. 024	-.003	-.135	. 083	. 510	. 207	. 772	-.448	. 242	. 794	. 181	1									
2	. 297	. 028	. 409	. 535	. 295	-.433	-.204	-.640	. 583	-.236	-.641	-.324	-.490	1								
3	. 222	. 008	**. 858**	. 732	**. 874**	. 032	. 168	-.301	. 501	. 149	-.278	-.072	-.101	. 569	1							
4	-.120	. 010	**-.808**	-.552	**-.909**	-.292	-.289	-.105	-.178	-.288	-.128	-.090	-.241	-.047	**-.801**	1						
5	-.228	-.007	**. 887**	**. 986**	. 783	-.130	. 019	-.377	. 394	-.004	-.358	-.221	-.181	. 626	. 706	-.459	1					
6	-.084	-.017	**. 964**	**. 823**	**. 995**	. 185	. 225	-.032	. 237	. 216	-.006	-.013	. 149	. 209	**. 839**	**-.920**	. 749	1				
7	-.056	. 019	-.695	-.415	**-.821**	-.369	-.309	-.249	-.033	-.315	-.272	-.154	-.355	. 188	-.658	**. 967**	-.297	**-.855**	1			
8	-.241	-.009	. 668	. 717	. 588	. 084	. 231	-.230	. 460	. 206	-.213	. 137	-.322	. 395	. 539	-.365	. 695	. 553	-.253	1		
9	-.071	-.013	**. 926**	**. 848**	**. 930**	. 028	. 085	-.151	. 212	. 074	-.127	-.183	. 131	. 321	. 792	-.799	**. 801**	**. 930**	-.714	. 403	1	

slo	. 081	. 143	-.035	-.035	-.036	. 039	. 037	. 020	. 059	. 038	. 022	. 053	. 008	. 145	. 077	. 028	-.010	-.048	. 061	. 012	-.058	1

1. bio1, Annual mean temperature; 2. bio2, Mean diurnal range; 3. bio3, Isothermality; 4. bio4, Temperature seasonality; 5. bio5, Max temperature of warmest month; 6. bio6, Min temperature of coldest month; 7. bio7, Annual temperature range; 8. bio8, Mean temperature of wettest quarter; 9. bio9, Mean temperature of driest quarter; 10. bio10, Mean temperature of warmest quarter; 11. bio11, Mean temperature of coldest quarter; 12. bio12, Annual precipitation; 13. bio13, Precipitation of wettest month; 14. bio14, Precipitation of driest month; 15. bio15, Precipitation seasonality; 16. bio16, Precipitation of wettest quarter; 17. bio17, Precipitation of driest quarter; 18. bio18, Precipitation of warmest quarter; 19. bio19, Precipitation of coldest quarter; alt: altitude; slo: slope; asp:aspect.

Table S5. The relevant parameters for the best model.

settings	FC	RM	train.AUC	avg.test.AUC	AICc	delta.AICc
PHT_1.5	PHT	1.5	0.9052	0.8975	7587.4064	0
